# A mechanism of airway injury in an epithelial model of mucociliary clearance

**DOI:** 10.1186/1465-9921-5-10

**Published:** 2004-08-24

**Authors:** Darryl W O'Brien, Melanie I Morris, Jie Ding, J Gustavo Zayas, Shusheng Tai, Malcolm King

**Affiliations:** 1 Pulmonary Research Group, Department of Medicine, University of Alberta, Edmonton, Alberta, Canada

## Abstract

We studied the action of sodium metabisulphite on mucociliary transport in a frog palate epithelial injury model, hypothesizing that it may be useful for the study of mechanisms of airway injury. Sodium metabisulphite (MB) releases SO_2 _on contact with water. SO_2 _is a pollutant in automobile fumes and may play a role in the exacerbation of airway disease symptoms. We first investigated its effect on mucociliary clearance. MB 10^-1 ^M, increased mucociliary clearance time (MCT) by 254.5 ± 57.3% of control values, (p < 0.001, n = 7). MB 10^-4 ^and 10^-2 ^M did not interfere with mucus clearance time compared to control values. In MB-treated frog palates, MCT did not return to control values after one hour (control, 97.3 ± 6.3% vs. MB, 140.9 ± 46.3%, p < 0.001, n = 7). Scanning EM images of epithelial tissue were morphometrically analyzed and showed a 25 ± 12% loss of ciliated cells in MB palates compared to controls with an intact ciliary blanket. Intact cells or groups of ciliated cells were found in scanning EM micrographs of mucus from MB-treated palates. This was associated with increased matrix metalloproteinase (MMP-9) activity in epithelial tissue and mucus. We suggest that the loss of ciliated cells as a result of MMP-9 activation prevented full recovery of MCT after MB 10^-1 ^M. The mechanism of action may be on epithelial cell-cell or cell-matrix attachments leading to cell loss and a disruption of MCT. Further studies are warranted to determine whether this is an inflammatory mediated response or the result of a direct action on epithelial cells and what role this mechanism may play in the progression to chronic airway diseases with impaired mucociliary clearance.

## Background

Particle clearance in the airways is dependant on mucus and cilia [1]. The cilia beat frequency, mucus secretion rate and the properties of mucus are variables important in normal and effective mucociliary clearance [2]. However, the study of mucociliary clearance in intact mammalian airways in humans or small mammals is technically difficult. It is worthwhile, therefore, to develop alternate models that, by way of ease of preparation and homology to human conductive airways, can yield important knowledge in understanding the basic mechanisms involved in airway diseases. The bullfrog palate provides an excellent integrated model system for studying all the relevant variables for mucociliary clearance including mucus secretion rate, cilia beat frequency, linear velocity of mucus, the viscoelastic properties of mucus and the transepithelial potential difference, indicative of changes in epithelial ion fluxes and water transport [2].

We have extended the physiological applications of the frog palate model to study the initial events of airway injury. To create an injury model from the fresh frog palate model, a solution of sodium metabisulphite was topically applied to the palate. Sodium metabisulphite has been shown to release sulfur dioxide (SO_2_) on contact with water and has been employed as an aerosol in other airway injury models to study hypersecretion and hyperplasia [2-6]. In dog studies, chronic exposure to SO_2 _produced symptoms similar to chronic bronchitis in humans [3].

We hypothesize that sodium metabisulphite will interfere with mucociliary clearance on the frog palate by disrupting the action of the ciliated epithelium, vital to the process of mucociliary clearance. The objective of this study was to evaluate the effect of sodium metabisulphite on mucociliary clearance on the frog palate. A further objective was to analyze tissue and mucus samples in ultra-structural and molecular studies to characterize the nature of the injury and to assess the potential involvement of matrix metalloproteinases which have been shown to play a role in airway injury and remodeling [7,8] and in cell-signaling pathways [14].

## Materials and Methods

### Development of a frog palate injury model

A fresh frog palate was prepared as previously described [1,2]. Briefly, the upper palate of the bullfrog (*Rana catesbiana*) was excised by cutting in the coronal plane from the lateral border of the mouth on one side of the head to the other. The excised palate was placed horizontally on gauze soaked in frog Ringers (2/3 Ringers + 1/3 distilled water, 207 mosml L^-1^) in a Petri dish. The palate was placed in an enclosed chamber (20 × 20 × 30 cm) maintained at a constant temperature (22–24°C) and continuously humidified at 100% with aerosolized frog Ringers generated by a Pari Jet^® ^nebulizer at a airflow rate of 8 L/min. The palate was allowed to stabilize for 15–20 min before any procedures were carried out on the palate.

Mucociliary clearance time (MCT) was measured by applying a droplet of mucus collected from the inferior (cut) edge of the palate that was placed at the superior edge of the palate near the midline. The action of cilia carries the mucus toward the inferior edge. The effect of various concentrations of sodium metabisulphite on MCT was measured following topical application on the palate. Frog Ringers was used as a control solution and vehicle for sodium metabisulphite. The volume of solution (either frog Ringers or sodium metabisulphite) that was applied to each palate was normalized among different sized palates according to the area of the palates surface. The area of the palate was approximated by measuring across the lateral-most borders of the jaw at the base of the palate, and calculating the area of the equivalent half-circle. The volume of solution applied was normalized to the area of each palate as shown: area = 3.5 cm^2 ^(volume applied = 2 μl), 4.5 (3 μl) 5.5 (4 μl) to 6.5 cm^2 ^(5 μl).

**Figure 1:**
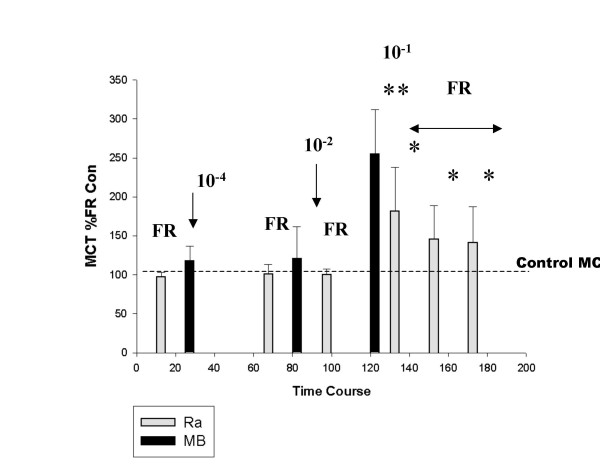
The effect of sodium metabisulphite on mucociliary clearance time (MCT). The results of seven independent experiments performed on seven different frog palates are shown in real time as displayed on the x-axis of the graph. Application of frog Ringers (FR) is shown by grey bars, while black bars indicate the application of sodium metabisulphite shown by the concentration (10^-4^, 10^-2 ^or 10^-1 ^M).

Using bromophenol blue in frog Ringers applied to the palate, it was shown that within two minutes of application, the solution was carried from the superior edge of the palate to the inferior edge by ciliary action. Therefore, when frog Ringers was applied to the palate, two minutes was allowed for the droplet of solution to disperse on the palate. This was followed by the measurement of MCT using a drop of frog mucus collected off the inferior (cut edge) of the palate and marked with carbon particles to enhance its visibility on the palate surface. The movement of the mucus droplet down the palate by ciliary action was observed through a stereomicroscope with a reticulated eyepiece and timed over a set distance of 4 mm, once it reached a steady speed. For each solution tested, five consecutive mucus clearance times were recorded and the average was used as the time point for that particular group of recordings. After a recovery period, sodium metabisulphite 10^-4 ^M was applied to the palate. After two minutes, another five measurements of MCT were recorded followed by a recovery period. At this point in time (70 min, shown in Figure 1), frog Ringers was applied and MCT was measured again. If this value was within 10% of the first application, the palate was considered to have recovered back to the control condition. Sodium metabisulphite 10^-2 ^M (at 80 min) was applied followed by the measurement of MCT again. This was followed with a recovery period with the measurement of frog Ringers MCT again, which was shown to be not different from the previous controls.

Frog Ringers following each recovery period also represented a timed control prior to each dose of metabisulphite. However, in order to control for deterioration of palate over the course of the experiment, three sets of frog Ringers controls measured before the application of sodium metabisulphite 10 ^-1 ^M were plotted versus time and a line of best fit was determined (data not shown). No change in the significance of the slope of the line (equal to or close to '0') indicated that no significant deterioration of the palate had taken place over time. Within each individual experiment there was, however, some variability in controls. Therefore the control mucociliary clearance times that were used to determine a line of best fit (taken as 100%) were compared to the actual MCT measured at that particular point. Thus for each experiment the actual MCT was expressed as a percentage of the line of best fit of the control time which was extrapolated to the time of application of frog Ringers or sodium metabisulphite to the palate as shown ((actual MCT/predicted control MCT) ×100). Thus, there is variation within and between controls that are shown as a standard deviation for each time point (representing seven independent frog palate experiments).

MCTs for metabisulphite were expressed as a percentage of the line of best fit for frog Ringers controls, extrapolated to the time metabisulphite was applied. An increase in MCT compared to control times, indicated a slowing of the mucociliary clearance time. A minimum of fifteen minutes was allowed after metabisulphite, for MCT to return to the normal range, i.e. within 10% of the frog Ringers MCT, measured prior to metabisulphite. If recovery of MCT had not occurred after twenty minutes to within the range specified, frog Ringers was re-applied and the recovery period was repeated.

### Injury to the palate

A 50% increase in MCT was established *a priori *as indicative of a quantifiable injury to the mucociliary clearance system. Sodium metabisulphite was applied in progressively increasing concentrations from 10^-4^, 10^-2 ^and 10^-1 ^M. Each test solution was alternated with frog Ringers. A higher concentration of metabisulphite was not applied until the MCT had returned to within 10% of the previously measured frog Ringers control. In several experiments, pH was measured on the surface of the frog palate, using a solid-state micro pH electrode (Lazar Research Laboratories, Los Angeles, CA) connected to an Accumet^® ^pH meter (Model 925, Fisher Scientific, Nepean, ON, Canada) to continuously monitor changes on the palate surface during the application of sodium metabisulphite.

### Scanning electron microscope (SEM) studies

Samples of frog palate epithelial tissue and mucus were placed in 2.5% glutaraldehyde solution, immediately after collection and kept in a refrigerator at 4°C until processing. Samples were prepared for the SEM by standard methodology. Briefly samples were post-fixed in 1% osmium tetroxide in Milonig's buffer at room temperature for one hour. They were then washed briefly in distilled water and dehydrated in an increasing series of ethanol (50, 80 and 100%), ten minutes at each concentration, followed by two additional periods of absolute ethanol. The samples were further dehydrated by critical point drying at 31°C for 5–10 minutes, and then mounted on a specimen holder for drying overnight in a desiccator. In the final stage of preparation before viewing, the samples were sputter coated with gold (Edwards, Model S150B Sputter Coater) and examined with a Hitachi 2500S scanning electron microscope. High-resolution digital images were acquired directly to a computer for storage and reproduction.

### Morphometry

To quantify the area of cilia loss in fields of view in the electron microscope studies, image files were analyzed using Sigma Scan^® ^image analysis software to trace areas of cell loss and determine the areas of loss relative to the field of view. Fifteen fields from 3 samples exposed to sodium metabisulphite 10^-1 ^M were examined as well as samples from control tissue (exposed only to frog Ringers).

### Gelatinase zymography

Samples of frog palate epithelial tissue were removed following mucus clearance studies, snap frozen in liquid nitrogen and stored at -80°C until they were prepared for zymography. At that time the tissue samples were ground with a mortar and pestle to a powder, adding liquid nitrogen to the mort to keep the tissue frozen. Homogenization buffer (KCl, ZnCl_2_, EDTA and Tris-HCl) was added to the samples that were sonicated for 30 seconds and then centrifuged at 14,000 rpm for 15 minutes. The supernatant was collected and an aliquot removed for protein assay (BCA protein assay kit, PIERCE).

A 10 μl sample, normalized for protein content, was loaded on a separating gel (acryl amide and gelatin) and run at 120 volts for one hour. After electrophoresis, the gel was washed for one hour in 25% Triton-X100 at room temperature followed by incubation overnight in zymography development buffer (0.15 M NaCl, 0.5 mM CaCl_2_, 0.05% Azide NaN_3_, 50 mM Tris-Cl, 2 M Tris-HCl). The gel was then stained for 2 hours with 0.05 % Coomassie blue (R-250) in methanol: acetic acid: water (2.5:1:6.5) followed by de-staining in 20% isopropanol in 4% ethanol and 8% acetic acid. The presence of gelatinases (MMP 2 and 9) was shown by clear bands (no staining) corresponding to MMP standards (MMP 2 and 9) run in leftmost lane on the gel. Optical density was measured in a Bio-Rad Scanning densitometer.

### Statistical treatment of data

All measurements were expressed as mean ± standard deviation. Overall significance of the MCT results were tested using a one-way analysis of variance in SPSS, with differences among groups (of more than two) evaluated using planned orthogonal comparisons. For comparisons between two groups (density comparisons between control and MB in zymograms), a Student T-test was used. The level of significance was set at p < 0.05.

## Results

Figure 1 shows the effect of sodium metabisulphite on the MCT expressed as a percent of frog Ringers control times. MCT is shown for frog Ringers, sodium metabisulphite 10^-4^, 10^-2 ^and 10^-1 ^M and 3 consecutive recovery periods following sodium metabisulphite 10^-1 ^M in which frog Ringers was applied to the palate in twenty-minute intervals followed by a measurement of MCT. The average frog Ringers MCT (in 7 frogs) measured initially at 15 minutes following an initial stabilization period was 97.3 ± 6.3 %. No difference in MCT was measured after the application of sodium metabisulphite 10^-4 ^(30 min) and 10^-2 ^M (60 min); whereas 10^-1 ^M sodium metabisulphite (at 100 min) increased MCT by 254.5 ± 57.3% compared to Ringers control MCT (taken as ~100%). Between 10^-4 ^and 10^-2 ^M sodium metabisulphite, there was no significant difference compared to control MCTs. This is illustrated by the dotted line in Figure 1. However, twenty minutes after sodium metabisulphite 10^-1 ^M, frog Ringers was applied but MCT did not recover to previous frog Ringers control times. Another twenty minutes of recovery was allowed and frog Ringers MCT was still not recovered. After an additional twenty minutes, frog Ringers MCT was measured for the third consecutive time, showing that after one hour of recovery (~170 min in the time course of the experiment), the MCT was still significantly different from the initial frog Ringers MCT (140.9 ± 46.3 vs. 97.3 ± 6.3%, p < 0.001, n = 7).

**Figure 2:**
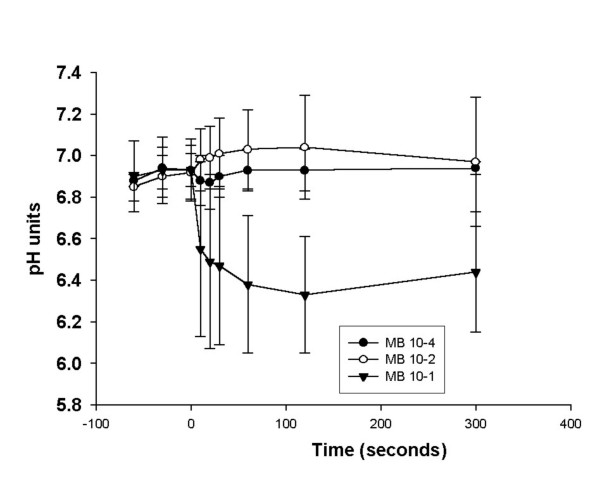
The effect of sodium metabisulphite on the pH, measured continuously on the surface of the palate, is shown for before and after three concentrations of sodium metabisulphite were applied (vol = 5 μl) to the palate.

MCT was significantly increased after sodium metabisulphite 10^-1 ^M. To determine if this acute effect was due to pH changes, possibly representing altered ion fluxes in the tissue, a micro pH electrode was placed on the palate to measure pH before and after the application of metabisulphite to the palate surface. This is shown in Figure 2. Prior to metabisulphite, the pH on the surface was 6.8–7.0 units. The pH was not significantly altered after the application of sodium metabisulphite 10^-4 ^and 10^-2 ^M. However, after sodium metabisulphite 10^-1 ^M, the pH declined within seconds, reaching a nadir at ~60 seconds. After 300 seconds, there was some recovery toward normal, but the pH was still 0.3–0.5 units below the initial control value. As shown in Figure 1, MCT recovered somewhat by 20 minutes after sodium metabisulphite 10^-1 ^M and showed continued (but incomplete) recovery after one hour. No corresponding pH measurements were taken at these time points.

### Scanning electron microscope studies

**Figure 3:**
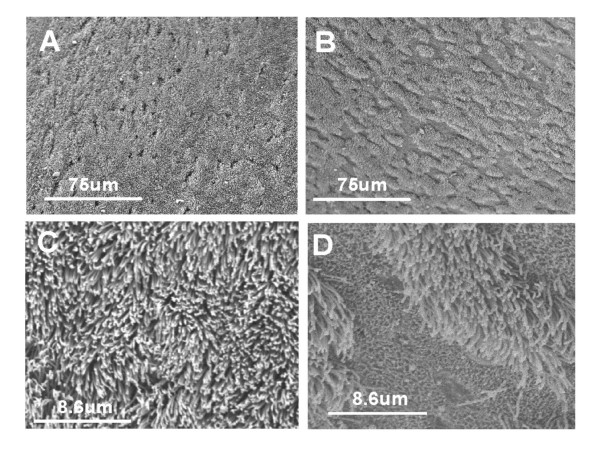
Scanning electron micrographs of control and MB-treated palates at a magnification of 400× (panels A and B respectively) and at 3500× (panels C and D respectively). In panel A, the ciliated epithelium completely covers the surface of the palate except where the openings to secretory cells are seen. In panel B, it can be seen that the ciliated surface is not continuous, but punctuated with numerous spaces where ciliated cells are not present. Panel C shows the high density of cilia on the palate surface, which under normal transport conditions, beat in a metachronal pattern to move a mucus layer over them. In panel D, the continuity of the ciliated layer is interrupted by spaces where ciliated epithelial cells are no longer present.

In Figure 3, Panel A (X400) shows the normal cilia blanket, with pores of secretory cells visible. Panel B (X400) shows regions of the palate surface devoid of cilia after 10^-1 ^M sodium metabisulphite was applied. The normal continuous covering of cilia is shown greater detail in Panel C (x3500) and in Panel D after 10^-1 ^M sodium metabisulphite, where a region of exfoliation is shown more clearly at the higher magnification. The absence of cilia and ciliated epithelial cells is visible, with only the extracellular matrix remaining. Morphometry to quantify the area of exfoliation determined in a five different field from three independent experiments, revealed that after sodium metabisulphite 10^-1 ^M, there was a 25 ± 11.8% loss of ciliated epithelial cells from these palates compared to none in control palates.

**Figure 4:**
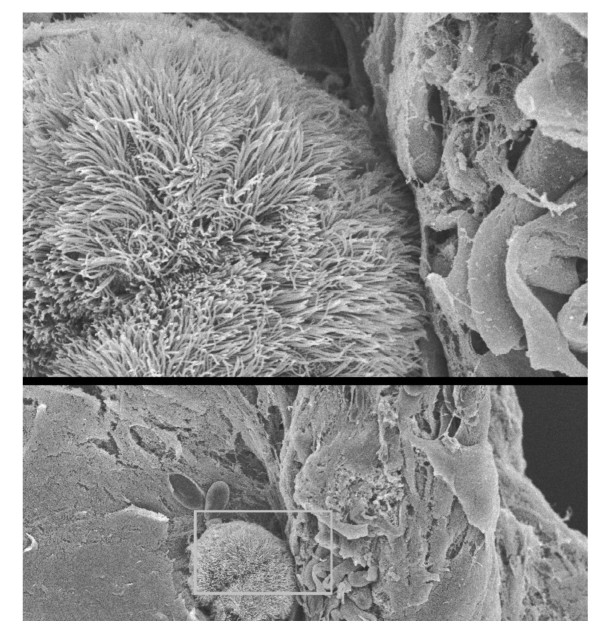
A sample of mucus taken off the palate after MB treatment showed groups of intact ciliated cells. This would suggest that the cells, which were exfoliated from the epithelial surface, were carried off the palate in the mucus layer by the process of mucociliary clearance.

SEM of the palate surface following sodium metabisulphite 10^-4 ^and 10^-2 ^M, showed no ultra structural changes compared to control palates to which frog Ringers had been applied. Figure 4 shows a split micrograph of mucus collected from a palate after sodium metabisulphite 10^-1 ^M. At lower power (X400) a grouping of ciliated cells are visible in the mucus. At the higher power (x2000), intact ciliated epithelial cells are clearly shown.

### Gelatinase zymography

**Figure 5:**
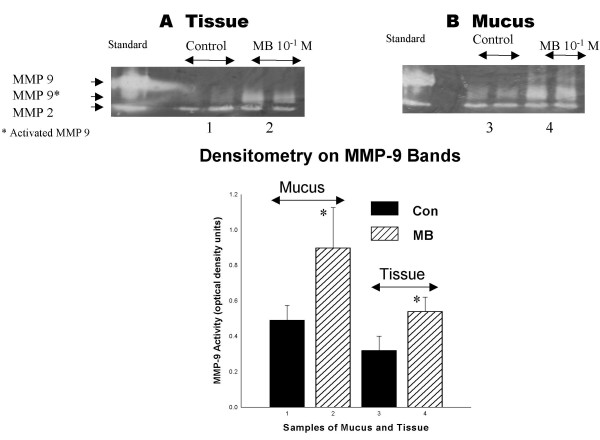
Representative zymograms from tissue (A) and mucus (B) shows the standards for MMP9 (top band, ~92 kD, latent size) and MMP2 (lower band, ~72 kD, latent size) in the leftmost lane. To the right of standard in each zymogram, two sets of bands are visible, corresponding to MMP-9 and MMP-2 levels of activity in duplicate samples of control tissue. In the next two lanes are duplicate sets of bands from an experiment which shows increased activated MMP-9 and possibly MMP-2 activity in sodium metabisulphite 10^-1 ^M treated tissue in both tissue and mucus. The bar graph only shows a comparison of the scanning density of the MMP-9 bands since the MMP-2 control and sodium metabisulphite-treated tissue showed similar activation. A significant increase in activated MMP-9 was seen in sodium metabisulphite-treated mucus and tissue (* p < 0.05, n = 3 for each).

**Figure 6:**
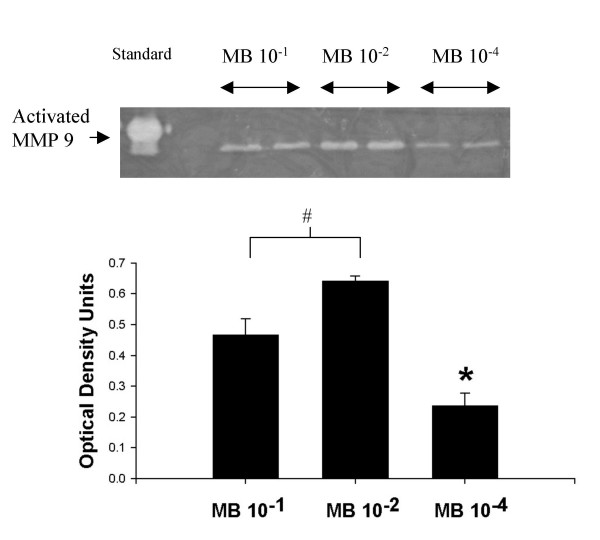
MMP-9 activation in palate tissue is a dose-related effect. The representative zymogram shows bands corresponding to MMP-9 activity in tissue samples treated with MB 10^-1^, 10^-2 ^and 10^-4 ^M. The MMP-2 bands have been removed from this gel as no differences were seen. Densitometry of the MMP-9 bands showed that MB 10^-1 ^M showed less activity than MB 10^-2 ^M, whereas MB 10^-4 ^M showed significantly less activation than either of the higher doses. The bar graph shows the average results in tissue from three separate experiments.

Figure 5 shows two representative zymograms from tissue and mucus. In 5A from tissue, in the left lane, two bands are visible representing MMP-9 (92 kD) and MMP-2 (72 kD) standards. In sodium metabisulphite 10^-1 ^M treated tissue (two rightmost columns), bands representing MMP 9 activity were seen whereas only faint bands were visible in control tissue. Statistical comparison of densitometry bands showed significant activation (p < 0.05, n = 3). MMP-2 activity (in the bottom row on the zymogram) may have also increased, but since control tissue showed similar activation these results are inconclusive. A similar state of MMP activation in mucus is shown in Figure 5B. Increased activated MMP-9 was observed in the mucus from metabisulphite-treated palates (p < 0.05, n = 3) compared to mucus from frog Ringers-treated palates. To test if MMP-9 activation was related to sodium metabisulphite concentration, samples of epithelial tissue were treated with sodium metabisulphite 10^-4^, 10^-2 ^and 10^-1 ^M, and prepared for zymography (Figure 6). Optical density analysis showed that activation of MMP-9 after sodium metabisulphite 10^-2 ^M was greater than after sodium metabisulphite 10^-1 ^M (#, p < 0.05, n = 3) while both were greater than MMP-9 activation following application of 10^-4 ^M sodium metabisulphite (*, p < 0.05, n = 3).

## Discussion

The important findings of this study are: 1. the development of a model of airway epithelial injury that can be used for study of ultra-structural and molecular events in airway injury that are directly related to the disruption of mucus clearance; 2. that sodium metabisulphite (by releasing SO_2 _on contact with water) has an acute effect on mucus clearance followed by incomplete recovery of mucus clearance time; 3. ultra-structural studies showed that areas of ciliated epithelial cells were lost from the palate surface resulting in an incomplete recovery of mucus clearance. Loss of cilia has been previously reported following exposure to SO_2 _in dogs [3]. The implication is that loss of cilia may affect mucus clearance in number of airway diseases. The mechanism of this effect requires further study for a more complete understanding of the events involved in this process. Intact ciliated epithelial cells were found in the mucus from 10^-1 ^M sodium metabisulphite-treated palates but not from frog Ringers-treated control palates; 4. Gelatinase zymography showed increased activity of MMP-9 after sodium metabisulphite (10^-4 ^to 10^-1 ^M) and this was shown to be a dose-related effect. It is noteworthy that gelatinase zymography showed increased activity of MMP-9 at each concentration of sodium metabisulphite, whereas ultrastructural damage was only found at the highest concentration; 5. The finding that intact ciliated cells were found in the mucus suggests that the action of activated gelatinases was on cell-cell or cell-matrix attachments resulting in the exfoliation of intact ciliated epithelial cells, which may have contributed to a slowing of mucus clearance over the surface of the palate. 

Additional studies are underway in our laboratory to identify possible the role of inflammatory mediators in the activation of matrix metalloproteinases in this model. Sodium metabisulphite may cause the release of oxidants or other mediators by epithelial cells [10,11] or from typical inflammatory cells, possibly activated neutrophils resident in the tissue, although the question of a time frame, related to neutrophil recruitment and activation would need to be clarified [12]. Oxidant products may cause activation of precursor forms of collagenase or gelatinase, leading to breakdown of the extracellular matrix [14]. It has been recently shown that mechanical stress resulted in the expression and release of gelatinases from epithelial and endothelial cells in the rat lung [7]. Further studies need to be undertaken to identify the source of MMP release following sodium metabisulphite and other airway modulating agents.

A high concentration of sodium metabisulphite may not be biologically relevant and represents a practical limitation to the applicability of the model. Nevertheless, a dose-response curve showed little effect on mucus clearance in the frog palate model at lower concentrations of sodium metabisulphite. Our findings suggest that this *ex vivo *model may be particularly useful in characterizing how an initial injury may be induced in ciliated epithelium. The ability to make functional measurements of mucociliary clearance in the *ex vivo *frog palate model allows for a correlation of variables in follow-up *in vitro *studies of tissue and mucus that may be interfering with mucociliary clearance.

Sodium metabisulphite, when applied to the palate is diluted in the periciliary fluid [9]. The dilution in palate surface fluid reduces the concentration of the applied metabisulphite. By approximating the area of the palate as one-half the area of a circle (~5 cm^2 ^on average) and assuming a mucus plus periciliary layer of 10 μm, a volume of 5 μl would effectively be diluted by as much as 1–2 orders of magnitude (assuming it spread over at least half the area of the palate). This calculation would suggest that sodium metabisulphite 10^-1 ^M was effectively and rapidly diluted to 10^-2 ^M or less. It follows that the lower concentrations of metabisulphite would be effectively less than the stock concentrations. Since, the effective concentration was determined experimentally in a dose-response experiment as that dose that produced a 50% or greater increase in the mucus clearance time, and since only the highest concentration of metabisulphite produced this effect, this concentration became physiological relevant to the outcome of these experiments. Lower concentrations (10^-4 ^and 10^-2 ^M) were also used, even though no effect on mucociliary clearance time was observed in the dose-response experiments, to determine if there might be some quantifiable effect at the cellular level, which was not manifested as a decrement in mucociliary clearance.

In several experiments the continuous pH response following the application of sodium metabisulphite 10^-1 ^M to the palate surface was monitored for 5 minutes. The pH measured on the palate prior to metabisulphite was 6.9 ± 1.4 units. There was a decrease in pH following sodium metabisulphite 10^-1 ^M, reaching a nadir of 6.4 ± 0.25 pH units after 60 seconds. Sodium metabisulphite 10^-4 ^and 10^-2 ^M did not cause any decrease in pH on the palate after application. Although the observed decrease in pH with sodium metabisulphite 10^-1 ^M is relatively minor (<0.5 pH units), it may have been sufficient to influence ion channels, possibly disrupting ciliary beating and causing chemical changes such as the induction of inflammatory mediators [5,15]. The dramatic increase in mucus clearance time seen 1–2 minutes after the application of MB 10^-1 ^M occurred in a similar time frame to the pH changes. After five minutes, the pH was returning toward normal, and within 20 minutes there was some recovery of mucus clearance time. An *in vitro *study [13] examined the effect of pH changes on ciliary beat frequency and found that the beat frequency was stable between 7.5 and 10.5 pH units. A significant decrease in beat frequency was noted at lower pH values. This report is consistent with our study that suggests that the transient decrease in pH caused a transient slowing or even cessation of ciliary beat frequency.

The increase seen in mucus clearance times after 10^-1 ^M sodium metabisulphite (~250% compared to control, ~100%) was followed by a recovery (120 to 170 min in Figure 1) to ~150% compared to control (still significantly different from control) nevertheless, demonstrated recovery from the acute response. It is possible that recovery could have been attenuated by the inability of the cilia to clear sodium metabisulphite off the palate. Alternately, SE micrographs showed that, in metabisulphite-treated palates, significant areas of exfoliation were present. It was shown by morphometric analysis that areas of the palate were devoid of ciliated cells, compared to an uninterrupted "carpet" of cilia in control palates. Although mucus continued to move across the palate, the loss of a significant portion of the ciliary layer, replaced by gaps in the ciliated surface, would contribute to a sustained (non-recoverable) increase in MCT. A further finding of intact, ciliated epithelial cells in mucus, recovered from metabisulphite-treated palates, suggested that exfoliation of intact ciliated cells may involve the action of proteases on cell-cell or cell matrix attachments. Gelatinase zymography showed increased activity of MMP-9 in tissue and mucus from metabisulphite-treated palates compared to controls.

## Conclusion

We have shown from the zymographic studies, taken together with the scanning electron microscope studies, that MMP-9 activation was associated with the loss of ciliated cells from the palate. These results suggest the sustained increase in MCT as measured directly on the frog palate may have been due to the action of sodium metabisulphite to activate MMP-9 leading to a loss of ciliated epithelial cells. How this occurs at the cellular level is a question that remains to be answered. Further studies that clarify a site of action of the MMPs and a source of MMPs in this model will be important to determine the mechanism of action of this effect. How MMPs are activated in the tissue is another important question. An understanding of this injury mechanism may lead to ways to intervene in the early stages of airway diseases with symptomatic signs of impaired of mucociliary clearance.
